# Vascular endothelial growth factor is an autocrine growth factor, signaling through neuropilin-1 in non-small cell lung cancer

**DOI:** 10.1186/s12943-015-0310-8

**Published:** 2015-02-20

**Authors:** Martin P Barr, Steven G Gray, Kathy Gately, Emily Hams, Padraic G Fallon, Anthony Mitchell Davies, Derek J Richard, Graham P Pidgeon, Kenneth J O’Byrne

**Affiliations:** Thoracic Oncology Research Group, School of Clinical Medicine, Institute of Molecular Medicine, Trinity Centre for Health Sciences, St. James’s Hospital & Trinity College Dublin, Dublin, Ireland; School of Medicine, Trinity Biomedical Sciences Institute, Trinity College Dublin, Dublin, Ireland; Irish National Centre for High Content Screening & Analysis, School of Clinical Medicine, Trinity College Dublin, Dublin, Ireland; Cancer & Ageing Research Program, Queensland University of Technology, Brisbane, Australia; Department of Surgery, Institute of Molecular Medicine, St James’s Hospital & Trinity College Dublin, Dublin, Ireland

**Keywords:** NSCLC, VEGF, Neuropilins, Survival, Cell signaling

## Abstract

**Background:**

The VEGF pathway has become an important therapeutic target in lung cancer, where VEGF has long been established as a potent pro-angiogenic growth factor expressed by many types of tumors. While Bevacizumab (Avastin) has proven successful in increasing the objective tumor response rate and in prolonging progression and overall survival in patients with NSCLC, the survival benefit is however relatively short and the majority of patients eventually relapse. The current use of tyrosine kinase inhibitors alone and in combination with chemotherapy has been underwhelming, highlighting an urgent need for new targeted therapies. In this study, we examined the mechanisms of VEGF-mediated survival in NSCLC cells and the role of the Neuropilin receptors in this process.

**Methods:**

NSCLC cells were screened for expression of VEGF and its receptors. The effects of recombinant VEGF and its blockade on lung tumor cell proliferation and cell cycle were examined. Phosphorylation of Akt and Erk1/2 proteins was examined by high content analysis and confocal microscopy. The effects of silencing VEGF on cell proliferation and survival signaling were also assessed. A Neuropilin-1 stable-transfected cell line was generated. Cell growth characteristics in addition to pAkt and pErk1/2 signaling were studied in response to VEGF and its blockade. Tumor growth studies were carried out in nude mice following subcutaneous injection of NP1 over-expressing cells.

**Results:**

Inhibition of the VEGF pathway with anti-VEGF and anti-VEGFR-2 antibodies or siRNA to VEGF, NP1 and NP2 resulted in growth inhibition of NP1 positive tumor cell lines associated with down-regulation of PI3K and MAPK kinase signaling. Stable transfection of NP1 negative cells with NP1 induced proliferation *in vitro*, which was further enhanced by exogenous VEGF. *In vivo*, NP1 over-expressing cells significantly increased tumor growth in xenografts compared to controls.

**Conclusions:**

Our data demonstrate that VEGF is an autocrine growth factor in NSCLC signaling, at least in part, through NP1. Targeting this VEGF receptor may offer potential as a novel therapeutic approach and also support the evaluation of the role of NP1 as a biomarker predicting sensitivity or resistance to VEGF and VEGFR-targeted therapies in the clinical arena.

**Electronic supplementary material:**

The online version of this article (doi:10.1186/s12943-015-0310-8) contains supplementary material, which is available to authorized users.

## Background

Despite improvements in conventional anti-cancer therapies such as chemotherapy, radiotherapy and surgery, the five-year survival for patients with non-small cell lung cancer (NSCLC) remains poor. Vascular endothelial growth factor (VEGF) is produced by most tumor types and stimulates the growth of new blood vessels within a tumor where it plays a pivotal role in the process of angiogenesis [1]. The biological effects of VEGF are mediated via binding to specific tyrosine kinase receptors including VEGFR-1 (Flt-1) and VEGFR-2 (KDR) in addition to non-tyrosine kinase receptors such as Neuropilin-1 (NP1) and Neuropilin-2 (NP2). Co-expression of NP1 and NP2 in NSCLC tissue is significantly correlated with tumor progression and poor prognosis [2]. NP1 has also been shown to be an independent predictor of cancer relapse and poor survival in NSCLC patients [3].

In Phase III trials, blocking VEGF using the recombinant humanized VEGF monoclonal antibody Bevacizumab (Avastin®) has proven successful in increasing the objective tumor response rate and in prolonging progression-free and overall survival in patients with NSCLC [4,5]. The survival benefit is however relatively short and the majority of patients eventually relapse. The current use of tyrosine kinase inhibitors alone and in combination with chemotherapy has been underwhelming [6] and the precise effects of removing VEGF from the circulation remains unclear. In a recent study [7], it was demonstrated that circulating and tumor VEGF-A and NP1 tumor protein expression could select for patients most likely to benefit from the addition of Bevacizumab to chemotherapy in advanced or metastatic gastric cancer patients. Patients with low baseline expression of NP1 showed a trend towards improved overall survival compared to patients with high NP1 expression. These studies suggest that NP1 may play an important role in VEGF-mediated signaling in the tumor cells themselves.

In this study we demonstrate that VEGF is an autocrine growth and cell survival factor for NSCLC cells, acting principally through the NP1 receptor, promoting lung tumor growth. The results indicate that NP1, in particular, should be evaluated as a predictive biomarker with levels of expression potentially defining those patients most likely to benefit from VEGF targeted therapies. Furthermore, NP1 may be a target for therapy in NSCLC and other tumors.

## Results

### NSCLC cells express the classical VEGF and Neuropilin receptors

**Figure 1:**
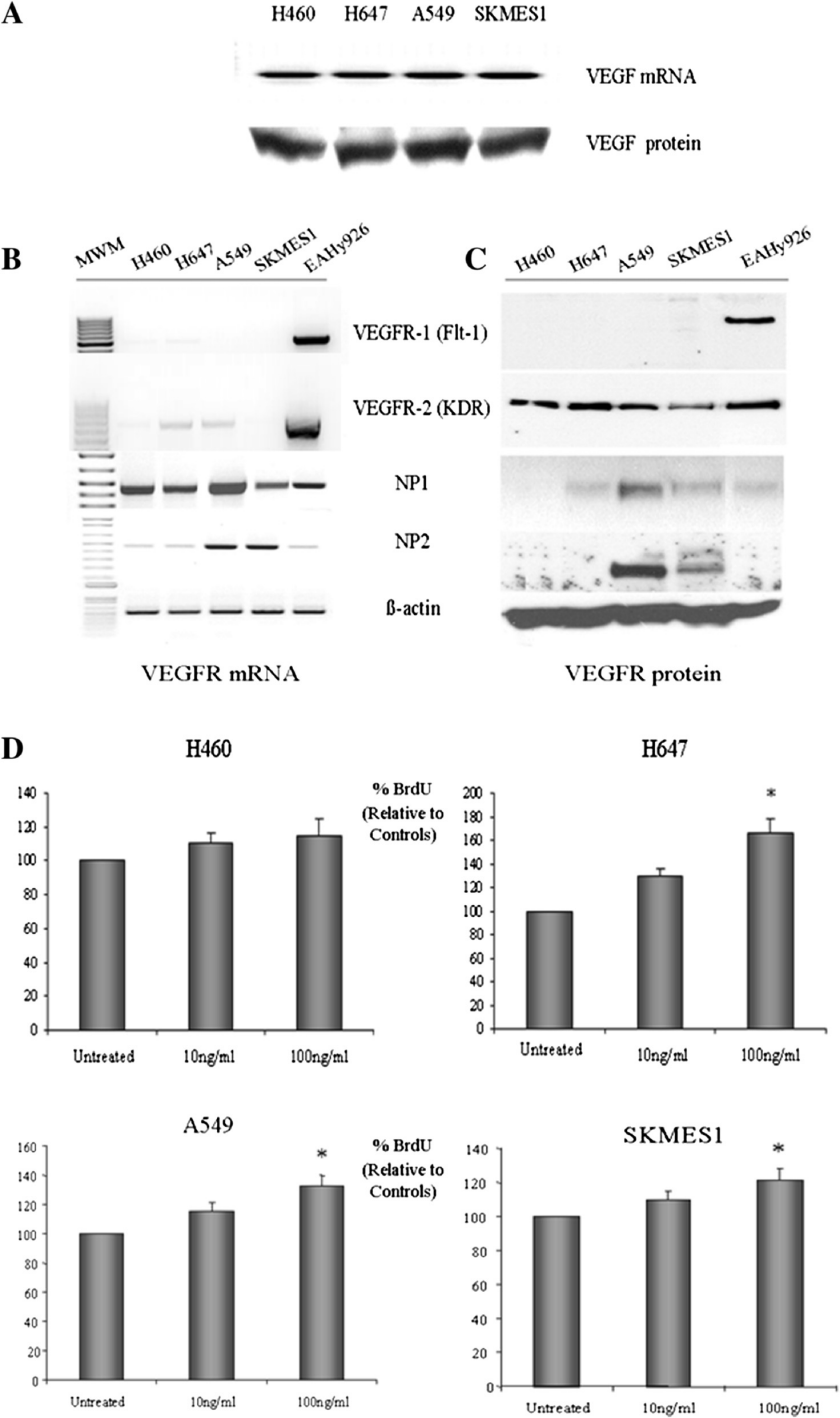
**VEGF**-**mediated survival of NSCLC cells.** Lung tumor cells were examined for their expression of VEGF mRNA and protein **(A)**. The VEGF receptors, VEGFR-1, VEGFR-2, NP1 and NP2 were also assessed at the mRNA **(B)** and protein **(C)** levels by RT-PCR and Western blot analysis, respectively. All four NSCLC cell lines were treated with recombinant human VEGF (100 ng/ml) for 48 h. Cell proliferation was then measured using the BrdU cell proliferation ELISA assay (*p < 0.05, VEGF vs untreated, n = 3) **(D)**.

A panel of NSCLC cell lines (H460, H647, A549 and SKMES1) was screened for the expression of the VEGF ligand (Figure 1A) and its receptors, VEGFR-1 (Flt-1), VEGFR-2 (KDR), NP1 and NP2 at the mRNA (Figure 1B) and protein levels (Figure 1C). VEGF_165_ mRNA and protein was expressed in all cell lines examined. While low levels of expression of VEGFR-1 and VEGFR-2 receptors were found at the mRNA level, NP1 and NP2 mRNA expression was more abundant in all cell lines examined. One may speculate that the low levels of VEGFR-1 and VEGFR-2 mRNA detected may be due to the lower sensitivity of RT-PCR over more sensitive and quantitative methods such as real-time PCR. However, despite the low levels of VEGFR-2 mRNA detected in these cells, significantly higher levels of VEGFR-2 protein were detected by western blot analysis. One possible explanation for this observation is that the VEGFR-2 protein is an inherently stable protein in these cells and does not undergo extensive degradation and/or recycling within the cell. As such, VEGFR-2 mRNA levels would be expected to be relatively low at steady-state levels and as such, reflect this low-turnover of protein. While all cell lines expressed VEGFR-2 at the protein level, VEGFR-1 protein was undetectable. NP1 protein expression was observed in all cell lines except for the H460 cell line, while NP2 protein was expressed in the adenocarcinoma A549 and squamous SKMES1 cells only.

### VEGF stimulates proliferation of Neuropilin-expressing NSCLC cells

VEGF_165_ is a potent mitogen for endothelial cells, mediating its biological effects via binding to its receptors VEGFR-1, VEGFR-2 and the NPs. Therefore, the effect of VEGF on the proliferation of NSCLC cells expressing different profiles of these receptors was measured following the treatment of cells with recombinant human VEGF (100 ng/ml) for 48 h (Figure 1D). VEGF stimulated the proliferation of NSCLC cells expressing the NP1 receptor (H647, A549 and SKMES1) with no effect on the NP1-negative cell line, H460. These findings demonstrate the role of VEGF_165_ in stimulating proliferation of NSCLC cells by interacting with the NP1 and/or NP2 receptors in the presence of the cell signal transduction receptor, VEGFR-2 (KDR).

### Neutralizing antibodies to VEGF inhibit proliferation of lung tumor cells

**Figure 2:**
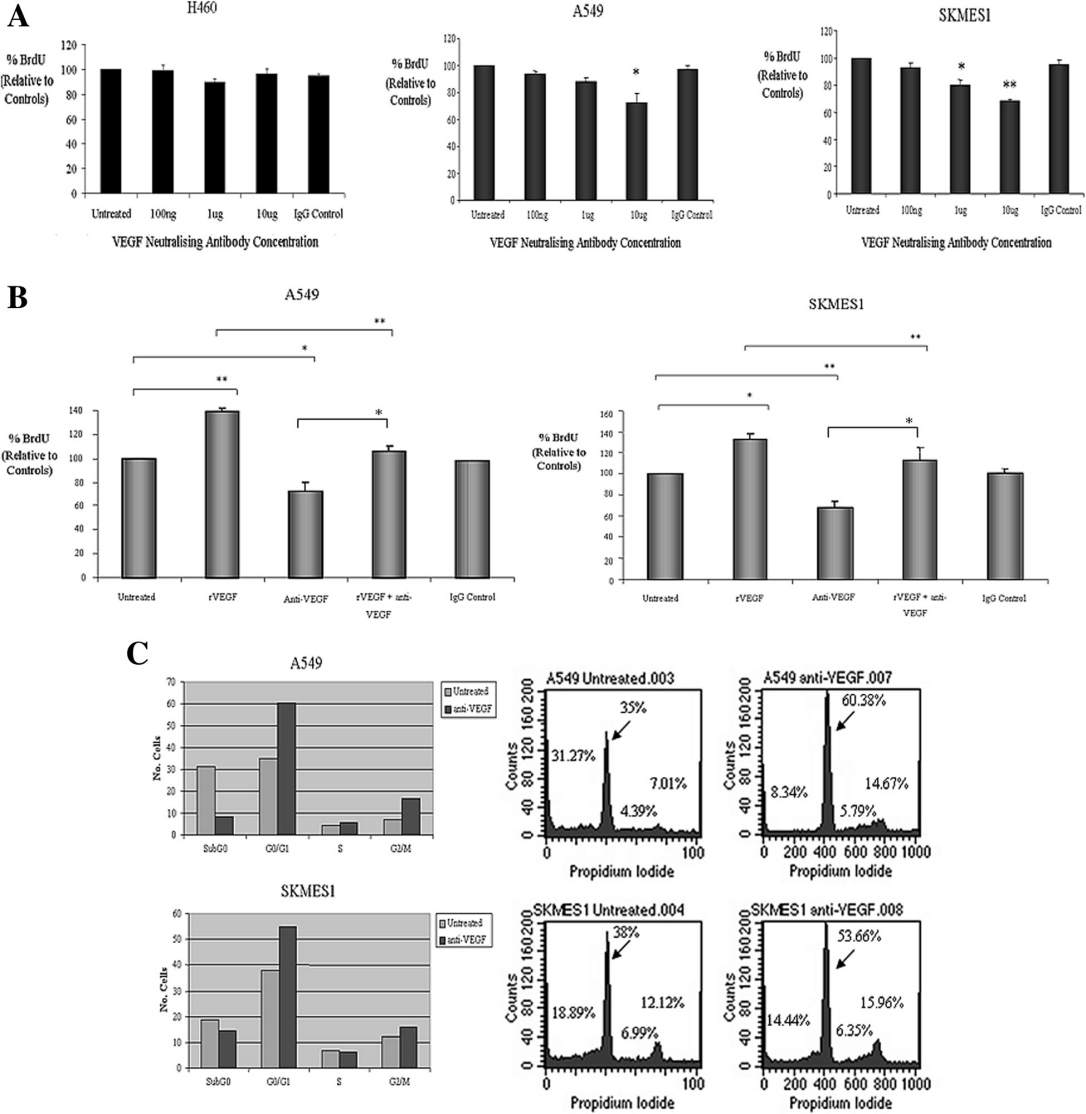
**VEGF blockade inhibits proliferation and induces cell cycle arrest of NSCLC cells.** Cells were treated with neutralizing antibodies to VEGF (100 ng/ml, 1 μg/ml, 10 μg/ml) for 48 h. An IgG isotype control was used as a control for antibody specificity. Cell proliferation was measured using the BrdU assay (*p < 0.05, **p < 0.01, VEGF neutralizing antibody vs untreated, n = 3) **(A)**. A549 and SKMES1 cells were treated with either recombinant VEGF (rVEGF), neutralizing antibodies to VEGF (Anti-VEGF), or both combined (A549 cells, *p < 0.05, rVEGF + anti-VEGF; *p < 0.01, untreated vs VEGF neutralizing antibody; **p < 0.001, untreated vs rVEGF, untreated vs rVEGF + Anti-VEGF, n = 3; SKMES1, *p < 0.05, rVEGF + anti-VEGF, *p < 0.01, untreated vs rVEGF; *p < 0.001, untreated vs Anti-VEGF, untreated vs rVEGF + Anti-VEGF, n = 3) **(B)**. To examine the effect of VEGF on cell cycle distribution, NSCLC cells were treated with neutralizing antibodies to VEGF (10 μg/ml) for 48 h. Cell cycle analysis was carried out (n = 2) by propidium idodide staining and examined by FACS **(C)**. Where indicated, data are expressed as the mean ± SEM from three independent experiments (n = 3). Statistical analysis was carried out by ANOVA using the Bonferroni multiple comparison post test.

The effect of neutralizing the biological activity of VEGF on cell proliferation was examined in the VEGF responsive cell lines A549 and SKMES1. Cells were treated with increasing concentrations of VEGF neutralizing antibodies (100 ng/ml-10 μg/ml) under reduced serum (0.5%) conditions for 48 h, after which time cell proliferation was measured. An IgG isotype control antibody was used to account for any non-specific effects of the antibody on cell proliferation. Neutralizing VEGF resulted in significant inhibition of cell proliferation in A549 and SKMES1 cells at 10 μg/ml. In addition, proliferation of SKMES1 cells in response to VEGF neutralization was also significantly decreased at 1 μg/ml (Figure 2A). We further examined whether the addition of VEGF was able to rescue cells following treatment with VEGF neutralizing antibodies. Whilst VEGF partially rescued cells from the effects of the monoclonal antibody, there was a statistically significant inhibition in proliferation of A549 and SKMES1 cells (Figure 2B) when treated concurrently with recombinant VEGF and neutralizing antibodies to VEGF, relative to the proliferative effects of VEGF alone. These results demonstrate that blocking VEGF inhibits VEGF-mediated proliferation of NSCLC cells.

### VEGF inhibition induces G0/G1 cell cycle arrest

In order to elucidate the mechanism underlying the inhibitory effect of VEGF neutralizing antibodies on lung tumor cell growth, changes in cell cycle distribution were examined. Relative to untreated control cells (A549; 35%, SKMES1; 38%), a significant accumulation of cells in the G0/G1 phase of the cell cycle was observed following VEGF neutralization (A549; 60.38%, SKMES1; 53.66%) (Figure 2C).

### VEGF mediates cell survival signaling of lung cancer cells via the PI3K and MAPK pathways

**Figure 3:**
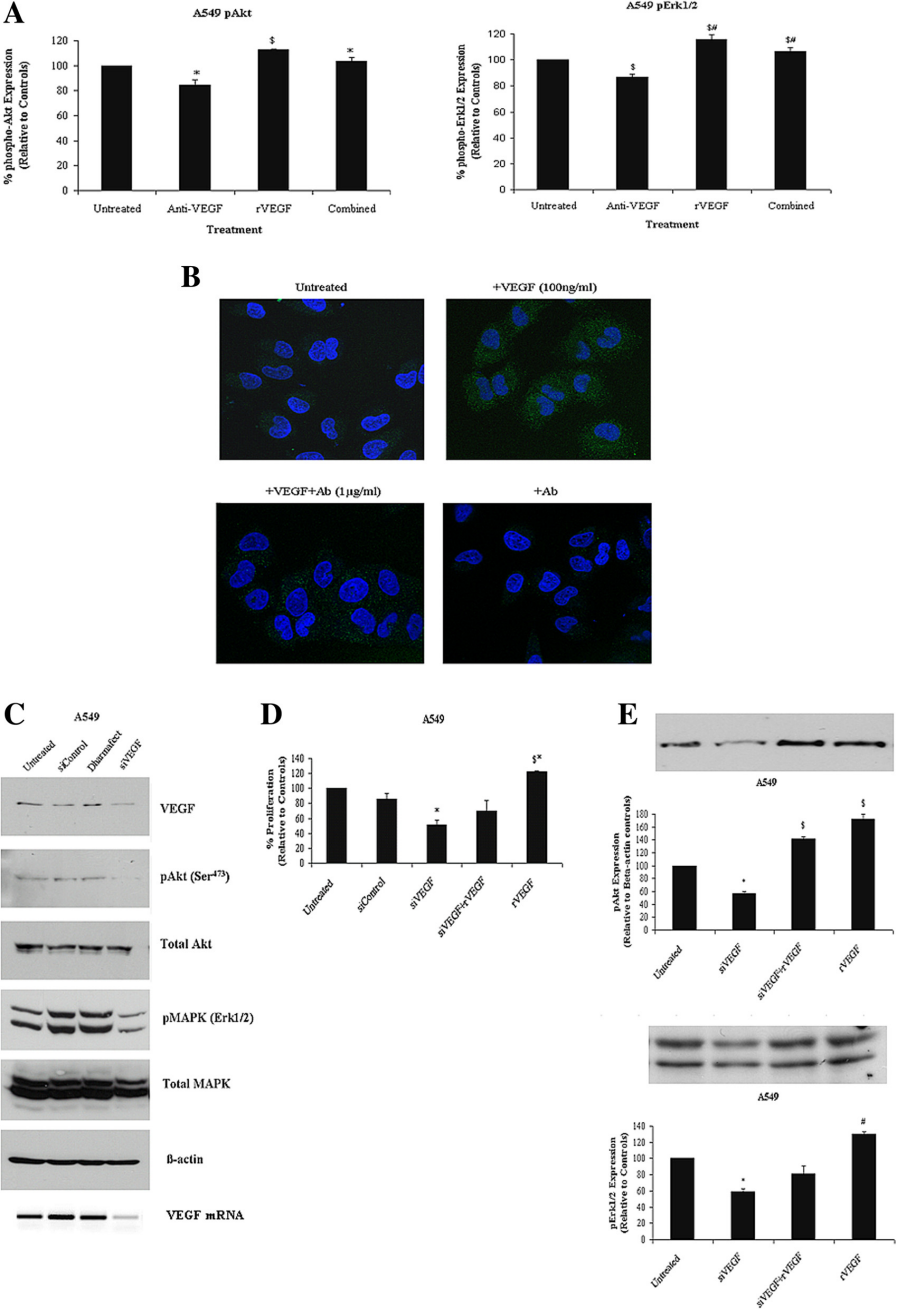
**PI3K and MAPK signaling pathways are stimulated by VEGF.** A549 cells were treated with recombinant human VEGF (100 ng/ml), VEGF neutralizing antibodies (1 μg/ml) or both combined. Phospho-Akt and phospho-Erk1/2 expression **(A)** and localization **(B)** were examined by high content screening (HCS) and confocal microscopy, respectively. Expression of the phosphorylated proteins, pAkt and pErk1/2, were quantified using IN Cell Analyzer 1000 Software (phospho-Akt; *p < 0.05 untreated vs anti-VEGF, anti-VEGF vs combined, ^$^p < 0.01 anti-VEGF vs rVEGF; phospho-Erk1/2; ^$^p < 0.01 untreated vs anti-VEGF, anti-VEGF vs combined, ^$#^p < 0.001 untreated vs rVEGF, anti-VEGF vs rVEGF, anti-VEGF vs combined, n = 3). Data are expressed as the mean ± SEM. Statistical analysis was carried out by ANOVA using the Bonferroni multiple comparison post test. Localization and expression levels of phospho-Akt and p44/p42 MAPK (Erk1/2) proteins were examined (×60 magnification) using a Zeiss LSM 510 laser scanning confocal microscope. Representative cells showing green fluorescence are indicative of phosphorylated Akt, while cell nuclei are stained blue. PI3-K and MAPK signaling proteins were examined by Western blot in response to siVEGF **(C)**. VEGF mRNA expression was also assessed by RT-PCR to confirm knockdown of VEGF. A549 cells were treated with siRNA to VEGF (100nM) or a scrambled siRNA control for 48 h, after which time, cell proliferation was measured **(D)** (*p < 0.05, $p < 0.01, ^#^p < 0.001, n = 3). Akt and MAPK (Erk1/2) phosphorylation was also examined in response to siVEGF **(E)**. Data are represented as the mean ± SEM. Statistical analysis was carried out by ANOVA using the Bonferroni multiple comparison post test (^#^p < 0.05, *p < 0.01, ^$^p < 0.001, n = 3).

NSCLC cells were treated with VEGF to examine its effect on the downstream signaling proteins of the phosphatidylinositol-3-kinase (PI3K) and mitogen-activated protein kinase (MAPK) signaling pathways using high content analysis (HCA), confocal microscopy and western blotting. Relative to untreated control cells, VEGF induced significant phosphorylation of phospho-Akt and phospho-MAPK (Erk1/2) in A549 (Figure 3A) and SKMES1 cells (Additional file 1: Figure S2). The expression of both proteins was observed mainly in the cytoplasmic compartment of both cell lines (Additional file 1: Figure S2). VEGF antibody significantly decreased phospho-Akt to below control levels. Similar results for phospho-Akt expression in response to VEGF and its blockade were also observed in SKMES1 cells. A significant increase in phospho-MAPK expression (Erk1/2) was observed in A549 and SKMES1 cells when treated with VEGF, while inhibiting VEGF significantly decreased VEGF-induction of pErk1/2. When cells were treated with both VEGF and VEGF neutralizing antibodies, in combination, VEGF-induced expression of pErk1/2 was significantly decreased. Confocal imaging of pAkt (Figure 3B) and pErk1/2 (Additional file 1: Figure S3. ) signaling proteins in response to VEGF and its blockade using neutralizing antibodies, demonstrated similar findings to those found by high content screening analysis.

### VEGF is an autocrine cell survival factor in non-small cell lung cancer

To assess the importance of VEGF on lung cancer cell survival, the effects of reducing VEGF expression in A549 and SKMES1 cells were examined. A549 cells were transfected with siRNA targeting the expression of VEGF (siVEGF), reducing the steady-state levels of VEGF mRNA by greater than 70%, as measured by RT-PCR and confirmed by Western blot (Figure 3C). A similar effect was also seen in SKMES1 lung cancer cells (Additional file 1: Figure S4.). The effect of decreased VEGF expression was examined on lung tumor cell growth relative to untreated cells and cells treated with scrambled controls. Significant inhibition of tumor cell proliferation was observed in A549 (Figure 3D) and SKMES1 (Additional file 1: Figure S4) cell lines. Phospho-Akt expression was significantly decreased in response to siVEGF in A549 and SKMES1 cells relative to untreated and scrambled (siControl) controls (Figure 3E). A similar effect was observed in the levels of expression of phospho-MAPK (Erk1/2) in A549 cells, but to a lesser extent in SKMES1 cells, in which case, decreasing VEGF did not significantly alter the MAPK pathway (Additional file a1: Figure S4). Addition of recombinant VEGF to siVEGF-treated cells significantly restored the expression of phospho-Akt to levels above that observed in siVEGF-treated cells alone in both cell lines. Importantly, the addition of VEGF to siVEGF-treated cells did not affect phospho-Erk1/2 expression levels in A549 or SKMES1 cells. These data further implicate the role of PI3K and, to a lesser extent, MAPK pathways in VEGF autocrine survival signaling in NSCLC.

### Gene silencing of the Neuropilin receptors abrogates tumor cell survival

**Figure 4:**
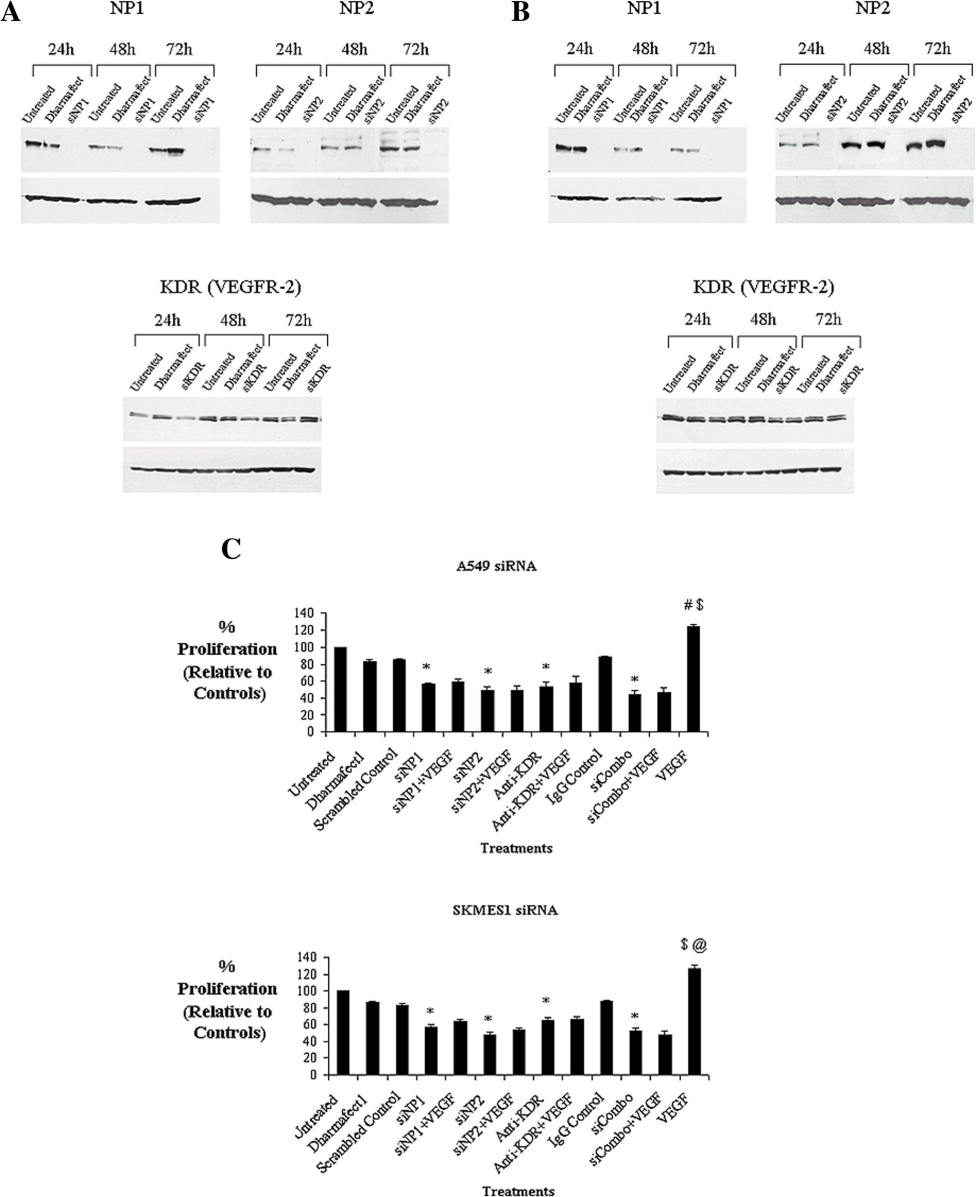
**Gene knockdown of VEGFR-** **2,** **NP1 and NP2 attenuates VEGF-** **mediated cell survival.** VEGFR siRNA was carried out in A549 **(A)** and SKMES1 **(B)** lung cancer cells over 24, 48 and 72 h and protein expression was examined by Western blot analysis. NSCLC cells were transfected with siNP1 (100 nM), siNP2 (100 nM), anti-KDR (10 μg/ml) antibody alone, and in combination, for 48 h. While an IgG isotype antibody was used as a control for antibody specificity for KDR, proliferation in response to each siRNA was measured relative to a scrambled control siRNA for siNP1 and siNP2. Exogenous recombinant VEGF (100 ng/ml) was added for a further 24 h following receptor knockdown/blockade, after which time, cell proliferation was measured **(C)** (*A549 cells*, *p < 0.001, control vs siNP1, siNP2, siCombo, anti-KDR; ^#^p < 0.05, control vs VEGF; ^$^p < 0.05, siNP1 + VEGF, siNP2 + VEGF, siCombo + VEGF, anti-KDR + VEGF vs VEGF alone, n = 3). (*SKMES1 cells*, *p < 0.001, control vs siNP1, siNP1, siCombo, anti-KDR, VEGF alone; ^$^p < 0.05, siNP1 + VEGF, siNP2 + VEGF, siCombo + VEGF vs VEGF alone; *p < 0.05, anti-KDR + VEGF vs VEGF alone, n = 3). Data are expressed as the mean ± SEM from three independent experiments. Statistical analysis was carried out by ANOVA using the Bonferroni multiple comparison post test.

To further extend our findings that NPs support VEGF autocrine survival signaling in NSCLC, a siRNA strategy was implemented to down-regulate the expression of the VEGF receptors in A549 (Figure 4A) and SKMES1 (Figure 4B) cells. NP1 and NP2 siRNA abrogated protein expression of both receptors at 24, 48 and 72 h post-transfection. In order to examine whether VEGF supports its autocrine function via VEGFR-2, cells were transfected with siKDR similar to that for siNP1 and siNP2. Due to low levels of KDR knockdown at 24-72 h, a neutralizing antibody approach was adopted to block VEGFR-2 (KDR). In order to elucidate the effect of VEGF on A549 and SKMES1 lung cancer cells upon knockdown/blockade of all three VEGF receptors (NP1, NP2, KDR), a combined siRNA (NP1, NP2) and receptor blockade (KDR) approach was used (siCombo) in the presence or absence of VEGF. In both A549 and SKMES1 cells, siNP1, siNP2 and KDR blockade alone, significantly inhibited tumor cell proliferation relative to untreated and scrambled controls. Knockdown/blockade of all three receptors significantly inhibited cell survival of both A549 and SKMES1 cells to levels below that for each receptor alone. However, when cells were treated with each siRNA and antibody in combination, the differences observed in proliferation were not statistically significant. Addition of recombinant VEGF was unable to rescue cells from the growth inhibitory effects of receptor blockade (Figure 4C), indicating that these receptors are critical for VEGF-mediated survival.

siNP1, siNP2 and KDR blockade significantly down-regulated phospho-Akt expression in A549 and SKMES1 cells (Additional file 1: Figure S5). Only NP1 knockdown significantly reduced pErk1/2 expression in A549 cells with a trend towards a decrease in pErk1/2 seen following treatment with siNP2 and anti-KDR. A non-significant decrease in pErk1/2 was evident following RNA inhibition and anti-KDR monoclonal antibody therapy in SKMES1 cells.

### NP1 promotes survival and constitutive PI3-kinase signaling of lung cancer cells and increases tumor growth in mice

**Figure 5:**
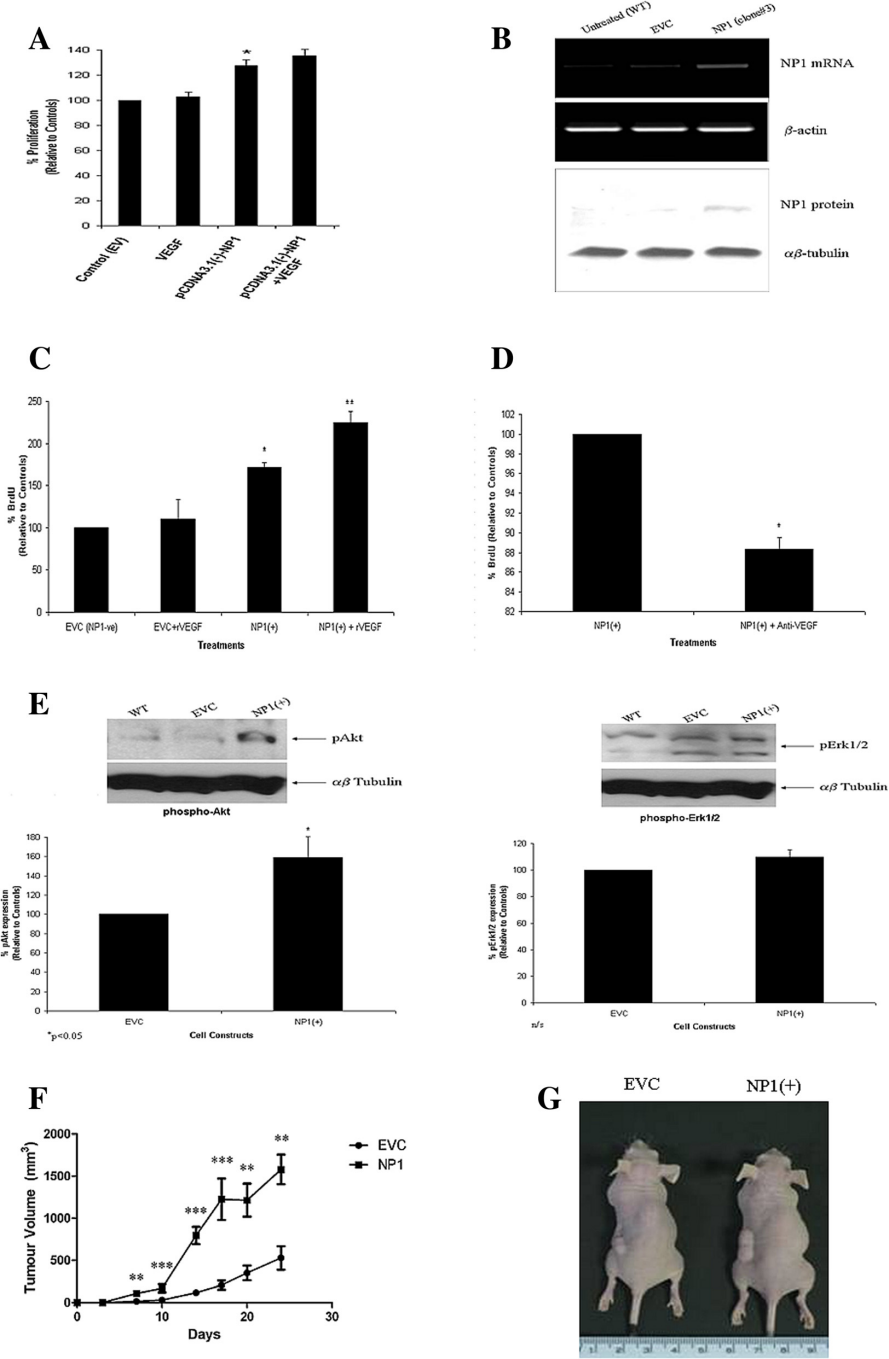
**H460 cells over**-**expressing NP1 promotes tumor growth in mice.** H460 cells (NP1-negative) were transiently transfected with a NP1 over-expression vector, pcDNA3.1(-)-NP1, or empty vector control, pcDNA3.1(-) for 48 h and examined for its effect on cellular proliferation in the presence or absence of VEGF (100 ng/ml) **(A)** (*p < 0.05 vs EVC). Validation of NP1 over-expression in stably transfected H460 cells was carried out at the mRNA and protein levels using RT-PCR and Western blot analysis, respectively **(B)**. The effect of VEGF stimulation on the proliferation of empty vector control cells and NP1 stably transfected cells were examined using the BrdU Cell Proliferation ELISA **(C)** (*p < 0.05, **p < 0.01). The response of NP1 over-expressing cells to VEGF blockade was examined using the BrdU Cell Proliferation ELISA following treatment with VEGF neutralizing antibodies **(D)** (*p < 0.05). In order to further confirm a role of the PI3-K and MAPK signaling pathways in VEGF-mediated NP1 survival signaling, protein expression of the downstream signaling proteins, phospho-Akt and phospho-Erk1/2, was examined by Western blot analysis **(E)** (*p < 0.05). Using an *in vivo* model, a tumor growth study was carried out using NP1 over-expressing H460 lung tumor cells in female nude mice. NP1 stably transfected H460 cells (3 × 10^6^), or empty vector control cells, were injected subcutaneously on the left-hand side dorsal flank of each mouse (n = 8/group). Tumor volumes were recorded every 3-4 days for 24 days **(F)**. From day 7 and up to day 24, by which time tumors had reached 2 cm^3^, lung tumor growth had increased significantly in mice injected with NP1 over-expressing cells (**p < 0.01; ***p < 0.001) compared to the much slower growing tumors observed in the control (EVC) group **(G)**. Data are represented as the mean ± SEM from three independent experiments **(A, C, D, and E)**. Statistical analysis for the *in vitro* analysis was carried out by ANOVA using the Bonferroni multiple comparison post test. For the xenograft study, a non-parametric Mann-Whitney Test was used.

The effect of NP1 over-expression on tumor cell proliferation/survival was examined in H460 cells which normally do not express this receptor. H460 cells were transiently transfected with a NP1 over-expression vector, pcDNA3.1(-)-NP1, and screened for its effects on cellular proliferation and/or survival of H460 NSCLC cells, compared to empty vector controls. Relative to controls (set at 100%), a significant increase in tumor cell growth was observed in H460 cells expressing the NP1 pcDNA3.1 vector for 48 h (128 ± 4.8% vs control) (Figure 5A). A stable transfectant was subsequently selected using antibiotic selection with G418 (Geneticin) at 800 μg/ml. Validation of NP1 over-expression was carried out at the mRNA and protein levels in the stably transfected cells (Figure 5B) and was found to be over-expressed relative to control cells. Similar to the effects observed for transient transfection, stable over-expression of NP1 had a significant increase in cellular proliferation at 72 h when compared to the empty vector controls (172 ± 5.2% vs control) (Figure 5C). The addition of exogenous VEGF had no effect on proliferation in cells transfected with the empty vector control, pcDNA3.1(-). In contrast however, VEGF stimulation of NP1 over-expressing cells induced a significant increase in cell proliferation relative to empty vector controls (224.5 ± 13.4% vs EVC control) and NP1 over-expressing cells alone (224.5 ± 13.4% vs 172 ± 5.2%). Blocking VEGF using neutralizing antibodies decreased proliferation of NP1 over-expressing cells compared to untreated cells (88.3 ± 1.2% vs untreated cells) (Figure 5D).

The effect of NP1 transfection on phosphorylation of the downstream signaling intermediates, Akt and Erk1/2 proteins was also examined. Compared to empty vector control cells, a significant increase in phosphorylated Akt was found in NP1 over-expressing cells (159 ± 7.5% vs EVC cells), but no change in levels of expression of phosphorylated Erk1/2 proteins (110 ± 5.4% vs EVC cells) (Figure 5E) was observed.

Based on these findings, and the effects of NP1 expression on lung tumor cell proliferation, an *in vivo* model was used to examine the effect of NP1 receptor over-expression on lung tumor growth. Following inoculation of cells, tumor growth was monitored every 3-4 days for 24 days post-injection into the flanks of athymic nude mice, and tumor volumes were recorded. A significant increase in lung tumor growth was observed from as early as day 10 compared to mice injected with control cells transfected with empty control vector. At day 24, by which time tumors had reached 2 cm^3^, lung tumor growth had increased significantly (**p < 0.01) (Figure 5F) in mice injected with NP1 over-expressing cells compared to the slower growing tumors observed in the control group (Figure 5G).

## Discussion

At present, drugs targeting angiogenic growth factors are postulated as mediating their anti-tumor effects by inhibiting new blood vessel formation. Experimental models have demonstrated that members of the VEGF family promote tumor growth *in vivo* by inducing angiogenesis [8]. When co-expressed in cells expressing VEGFR-2, NP1 enhances the binding of VEGF_165_ to VEGFR-2 and subsequent VEGF_165_-mediated chemotaxis [9,10]. Although the biological role of VEGFR-1 has remained unclear, cross-linking experiments have shown that VEGF_121_ is able to bind both NP1 and NP2 in cells that co-express VEGFR-1, suggesting an interaction between VEGFR-1 and the NPs [11]. Although experimental evidence indicates that endothelial migration and sprouting that is mediated by VEGF_121_ (which binds to both NP1 and VEGFR-2, but cannot form bridges between them) may be inhibited by anti-NP1 antibodies [12], it is possible that NP1 may have functions that are independent of VEGFR-2, potentially through the NP1 interacting protein (NIP) [13]. In xenograft experiments, anti-NP1 antibodies have a modest suppressive effect on tumor growth, but significant additive suppressive effects on tumor growth when combined with anti-VEGF therapies [14]. This is accompanied by reductions in tumor vascular density and maturity, suggesting that targeting NP1 is a valid anti-angiogenic strategy and may help overcome resistance to anti-VEGF therapies.

This anti-angiogenic hypothesis however fails to take into consideration that in patients, tumor cells may proliferate in the absence of neo-angiogenesis by co-opting and modifying the existing vasculature. A role for VEGF in preventing tumor cell apoptosis is supported by reports demonstrating that over-expression of the soluble VEGF receptor NP1, which prevents VEGF binding to the cell surface receptors in tumor cells, is associated with tumor cell apoptosis [15]. NP1 is expressed on many tumor cell types and increased expression of both NP1 and NP2 has been found to correlate with tumor aggressiveness, advanced disease and poor prognosis [16,17].

To address the hypothesis that VEGF is a growth and cell survival factor for NSCLC, cells were treated with VEGF_165_ that binds to all four VEGF receptors, VEGFR-1, VEGFR-2, NP1 and NP2. These data demonstrated that VEGF stimulated growth of lung tumor cells expressing NP1, but had no effect on cells that did not express the NP1 receptor. Of interest was our finding that H460 cells, in which NP1 receptor expression is absent, failed to respond to VEGF despite its expression of VEGFR-2. We believe that a critical element behind VEGF-mediated cell survival involves the Neuropilin receptors acting either as hetereodimers or homodimers. It was previously hypothesized by Soker *et al* [10] that in endothelial cells expressing both NP1 and VEGFR-2, NP1 mediates VEGFR-2 activity by serving as a co-receptor, thereby enhancing VEGF binding to the VEGFR-2 receptor resulting in VEGF-mediated downstream signaling events, chemotaxis and angiogenesis. As we currently know, Neuropilins are unable to form homodimers, and as such, must mediate their effects through heterodimeric interactions with other receptors such as KDR or Plexins, highlighting NP1, or NP2, as critical elements involved in mediating VEGF signaling and supporting cell survival in NSCLC.

In cells responding to recombinant VEGF protein, neutralizing antibodies to VEGF inhibited lung tumor cell growth and resulted in the arrest of cells in the G0/G1 phase of the cell cycle, suggesting an important role for VEGF signaling in lung tumor cells. Knockdown of VEGF expression in cancer cells which are responsive to VEGF, reduced cell proliferation further supporting a role for VEGF as a cell growth and survival factor in NSCLC. Such findings are in agreement with those previously reported [18] demonstrating a role for VEGF in the survival of H1299 lung cancer cells expressing VEGFR-1, VEGFR-2 and NP1. The effects of VEGF on downstream proliferation and survival signaling in the NP1 expressing cell lines, A549 and SKMES1, were clearly demonstrated in this study with induction of phosphorylation of the PI3K mediator Akt and, to a lesser extent, the MAPK signaling proteins Erk1/2, respectively.

These results and other accumulating evidence suggest that the function of VEGF in tumor progression may not be limited to angiogenesis and that the more important role of this pathway is in epithelial cell survival and proliferation [19]. VEGF autocrine signaling via NP1 has been demonstrated in breast cancer cells [20,21]. NP1 complexing with plexin-A1 involving both VEGF and SEMA3a has been implicated in the chemotaxis of breast cancer cell lines [22]. Preclinical data also support a role for tumor cell NP1 in mediating lung and renal cancer cell migration, proliferation and invasion [3,23]. In human FG pancreatic cells, over-expression of NP1 induced both Erk1/2 and JNK signaling pathways [24]. Down-regulation of the NP1 receptor using siRNA sensitized PANC-1 cells to the cytotoxic effects of the chemotherapeutic agent Gemcitabine, compared to NP1-over-expressing cells. Such findings further implicate NP1 as a cell survival factor in epithelial tumors. In prostate cancer, NP1 was found to be highly expressed by prostate cancer cell lines and displayed a positive association with invasiveness, suggesting that it may be one of the primary receptors responsible for VEGF autocrine effects in prostate cancer cells [25]. A positive association between NP1 expression and *in vivo* bone metastatic potential was found in ARCaPM xenografts and was further confirmed in clinical prostate cancer specimens. Hamerlik *et al* showed that VEGF-VEGFR2-NP1-mediated signaling in glioma stem-like cells is maintained in an autocrine manner through the continuous secretion of VEGF, thereby allowing constitutive activation of downstream pro-survival pathways and growth of glioblastomas, tumor invasion and increased resistance to treatment [26]. In other studies, VEGF and NP1 expression by tumor epithelial cells also regulates the stemness of cutaneous tumors and the expansion of the cancer stem cell (CSC) pool, contributing to enhanced tumor growth [27]. Conditional deletion of VEGF in tumor epithelial cells caused tumors to regress, whereas VEGF over-expression by tumor epithelial cells accelerated tumor growth. In addition, VEGF affected skin tumor growth by promoting cancer stemness and symmetric CSC division leading to CSC expansion. When expressed as a co-receptor in cutaneous CSCs, deletion of NP1 blocked the ability of VEGF to promote cancer stemness and renewal.

Our data support the observations that NPs play a central role in epithelial cancer cell survival. Stable transfection of NP1 in NP1 non-expressing cells induced NSCLC cell growth *in vitro* and *in vivo*. The *in vitro* proliferation was augmented by addition of recombinant VEGF. In contrast, stable knockdown of NP1, NP2 and anti-VEGFR-2 antibody treatment induced inhibition of tumor cell growth in NP1 positive lung adenocarcinoma and squamous cell lines. This work cannot discount an independent role for NP2 as a transmitter of cell survival signaling for VEGF and this will be explored in future studies. What is clear however, is that expression of NP1 together with VEGFR-2 may be critical for the autocrine survival and growth effects of VEGF in NSCLC.

The combined targeting of ligand and co-receptor may help to overcome resistance to targeted agents such as bevacizumab in a subset of patients demonstrated to over-express NP1. This is supported by preclinical data demonstrating that anti-NP1 antibodies have additive anti-cancer activity in combination with anti-VEGF therapy [14]. A more comprehensive analysis of the expression of NP1 by epithelial tumor cells may help to inform prospectively planned biomarker driven studies of the clinical benefit of bevacizumab, VEGFR- and NP1-targeted agents. In this regard, recent studies showing NP1 immuno-positivity by tumor cells in 6% of primary and 14% of metastatic breast cancers, and 36% of primary and 50% of metastatic NSCLC provides a framework for testing this combined approach in patients [28,29].

## Conclusions

This study demonstrates that VEGF is an autocrine growth factor for NP1 expressing NSCLC cells and may have important implications for the pathogenesis and treatment of NSCLC. These observations highlight the critical role of VEGF and its cognate receptors, in particular the Neuropilins, in the survival of lung cancer cells. VEGF is produced by many tumor cells. A number of studies have reported expression of NP1 in a variety of cancers such as prostate, pancreas, kidney, colorectal, brain, breast and liver cancer. Therefore, targeting this VEGF receptor may offer significant potential as a novel therapeutic approach and may change the way clinicians design studies aimed at targeting the VEGF survival pathway in cancer patients. Our results also support the evaluation of the role of NP1 as a biomarker predicting sensitivity or resistance to VEGF and VEGFR-targeted therapies in the clinical arena.

## Methods

### Cell lines

A panel of non-small cell lung cancer cells, H460 (large cell carcinoma), H647 (adenosquamous carcinoma), A549 (adenocarcinoma) and SKMES1 (squamous cell carcinoma) were used. H460 and H647 cells were purchased from the American Tissue Culture Collection (ATCC), while A549 and SKMES1 cells were purchased from the European Cell and Culture Collection (ECACC). H460 and H647 cells were maintained in Roswell Park Memorial Institute (RPMI-1640) medium in a humidified atmosphere of 5% CO_2_ in air at 37°C. A549 cells were maintained in Ham’s F12 supplemented with 4 mM L-glutamine, while SKMES1 cells were cultured in EMEM media supplemented with 2 mM L-glutamine and 1% non-essential amino acids (NEAA). All media were supplemented with 10% heat-inactivated fetal bovine serum (FBS), penicillin (100 U/ml) and streptomycin (100 μg/ml) (Lonza, UK). All cells were maintained as monolayer cultures and exponentially growing cultures were used in all experiments. All cell lines were tested and authenticated six months prior to this study using the PowerPlex® 16 HS System (Source BioScience, UK), a multiplex STR system.

### Analysis of mRNA expression by RT-PCR

Total RNA was isolated using Tri-reagent (MRC Inc, OH, USA). First-strand cDNA was prepared from 1 μg of total RNA using Superscript III reverse transcriptase (Invitrogen, UK) according to manufacturer’s instructions. PCR reactions were carried out for the VEGF receptors, VEGFR-1, VEGFR-2, NP1 and NP2. The endothelial cell line, EAhy926, was used as a positive control. Primer sequences used were as follows:

- VEGFR-1 Forward: 5′CAAGTGGCCAGAGGCATGGAGTT3′
- Reverse: 5′GATGTAGTCTTTACCATCCTGTTG3′
- VEGFR-2 Forward: 5′GAGGGCCTCTCATGGTGATTGT3′
- Reverse: 5′TGCCAGCAGTCCAGCATGGTCTG3′
- NP1 Forward: 5′ATGGAGAGGGGGCTGCCG3′
- Reverse: 5′CTATCGCGCTGTCGGTGTA3′
- NP2 Forward: 5′CCCCGAACCCAACCAGAAGA3′
- Reverse: 5′GAATGCCATCCCAGATGTCCA3′
- VEGF Forward: 5′CGCAAGCTTAGGAGTACCCTGATGAG3′
- Reverse: 5′CCGTCTAGAACATTTGTTGTGCTGT′
- β-actin amplification was carried out in parallel to account for loading differences between samples:
- β-actin Forward: 5′TGTTTGAGACCTTCAACACCC3′
- Reverse: 5′AGCACTGTGTTGGCGTACAG3′

Specificity of all primers was confirmed by comparing the primer sequence for each gene against the Genbank database. PCR products were analyzed on a 1% agarose gel and images acquired using the BioSpectrum® Imaging System (UVP, CA, USA).

### Western blot analysis

Total protein was extracted from cells using ice-cold RIPA buffer (50 mM Tris HCl, pH 7.4, 150 mM NaCl, 1 mM EDTA, 1% (v/v) Triton-X 100, 0.1% (w/v) SDS) supplemented with phenylmethylsulfonyl fluoride (PMSF) and protease inhibitor cocktail (2 mM AEBSF, 1 mM EDTA, 130 μM Bestatin, 14 μM E-64, 1 μM Leupeptin, 0.3 μM Aprotinin). Protein concentrations were determined using the bicinchoninic acid assay (BCA) as per manufacturer’s instructions. Protein (40 μg) from whole cell lysates was fractionated on 8% or 12% SDS-PAGE gels and transferred to a PVDF membrane (PALL Corporation, FL, USA). Transfer efficiency and loading was confirmed by reversible staining of the membrane with Ponceau S solution (Sigma-Aldrich, UK) following protein transfer. Membranes were blocked at room temperature with 5% non-fat dry milk in Tris-buffered saline (TBS) containing 0.1% Tween-20 (TBS-T), followed by incubation with the appropriate primary antibodies at room temperature or otherwise stated: rabbit anti-VEGF (Millipore, CA, USA), 1:2000 at 4°C in 5% BSA TBS-T (0.05%); mouse anti-Flt-1 (Millipore, CA, USA), 1:500 at 4°C in 3% Marvel TBS-T (0.05%); rabbit anti-KDR (Upstate, USA), 1:5000 at room temperature in 5% Marvel TBST-T (0.05%); goat anti-NP1 and rabbit anti-NP2 (Santa Cruz Biotech, CA, USA), 1:400 at room temperature in 5% Marvel TBS-T (0.1%); anti β-actin (Merck Biosciences, UK), 1:20000 at room temperature in 5% Marvel TBS-T (0.1%). Membranes were washed in TBST and incubated with a secondary horseradish peroxidase (HRP)-labeled antibody for 1 h at room temperature (1:2000 at room temperature in 0.1% TBS-T). Membranes were washed in TBST following incubation with secondary antibody. Bound antibody complexes were detected and visualized using SuperSignal™ West Pico enhanced chemiluminescence substrate (Pierce, IL, USA).

### Cell proliferation

Cell survival/proliferation was measured using the bromodeoxyuridine (BrdU) cell proliferation ELISA according to manufacturer’s instructions (Roche Diagnostics, Germany). BrdU labeling solution was added at a final concentration of 10 μM. Cells were fixed for 60 min followed by incubation for 90 min anti-BrdU antibody (1:100). Wells were washed and incubated in substrate solution. Absorbance was measured at 450 nm using a reference wavelength at 690 nm.

### High content imaging & confocal microscopy

NSCLC cells were seeded (1 × 10^4^) in MatriPlate™ 96-well glass bottomed micro-well plates (Matrical Bioscience, WA, USA) and allowed to adhere overnight. Following serum depletion (0.5% FBS), cells were treated with recombinant human VEGF (100 ng/ml), VEGF neutralizing antibodies (1 μg/ml) or in combination, for 6 h. Cells were fixed in 3% paraformaldehyde and washed in PBS. After washing, cells were blocked in 5% normal goat serum for 1 h followed by incubation with primary rabbit phospho-Akt (1:400) (Millipore) and phospho-p44/p42 MAPK (1:400) (Cell Signaling Technology) primary antibodies in 4% BSA overnight at 4°C. Cells were washed in blocking buffer and incubated with a secondary Alexa Fluor® 488 (Invitrogen) goat anti-rabbit antibody (1:1000), red phalloidin (1:1,000) and Hoechst 33342 (1:500) at room temperature for 1 h. After washing in PBS, localization and expression levels of phospho-Akt and phospho-Erk1/2 were examined on the In Cell 1000 analyzer (GE Healthcare, UK) using IN Cell Investigator high-content image analysis software (version 1.5). For confocal microscopy analysis, NSCLC cells were seeded in glass chamber slides and allowed to adhere overnight. Following serum depletion (0.5% FBS), cells were treated with recombinant human VEGF (100 ng/ml) or VEGF neutralizing antibodies (1 μg/ml) for 6 hrs. Cells were fixed in 3% paraformaldehyde and washed in PBS. After washing, cells were incubated in blocking buffer containing 5% bovine serum albumin (BSA) for 1 h and incubated with rabbit phospho-Akt (1:200) and p44/p42 MAPK (Erk1/2) (1:50) primary antibodies (Cell Signaling Technology) at 4°C overnight. Cells were then washed in PBS and incubated with a secondary Alexa Fluor® 488 (Invitrogen) goat anti-rabbit antibody (1:1000) and Hoechst 33342 for nuclear staining at room temperature for 1 h. After washing in PBS, localization and expression levels of phospho-Akt and phospho-Erk1/2 were examined using a Zeiss LSM 510 laser scanning confocal microscope (Carl Zeiss International, Germany).

### Cell cycle analysis

Cells were detached and pelleted by centrifugation at 1300 rpm for 3 min. Supernatants were discarded and cells were suspended in 1 ml phosphate-buffered saline (PBS) and fixed in 90% ice-cold ethanol. Following incubation at room temperature for 30 min, cells were resuspended in 1 ml PBS containing propidium iodide (25 μg/ml) and DNase-free RNase A (100 μg/ml) and left at 37°C for 30 min. DNA synthesis and cell cycle distribution was measured by FACS (Becton Dickinson, UK).

### siRNA transient transfections

siRNA ON-TARGETplus SMART pool siRNA for NP1, NP2, VEGF and VEGFR-2 (KDR) were designed and synthesized (Dharmacon Inc, USA). Each siRNA pool contains four individual sequences to silence target gene expression at the mRNA level by at least 75%. A non-targeting scrambled control was also included for each target gene of interest. Cells at 60% confluence were transfected in penicillin/streptomycin-free media with each siRNA (100 nM) using DharmaFect1 transfection reagent (Dharmacon Inc, USA) according to manufacturer’s instructions. After 6 h, siRNAs were removed and cells were maintained in complete media for 24, 48 and 72 h. At each time point, total protein was extracted from A549 and SKMES1 cells for Western blot analysis to determine knockdown of each gene at the protein level. As an alternative to siRNA, due to low levels of knockdown of VEGFR-2, a blocking antibody to VEGFR-2 (sc-19530) (Santa Cruz Biotech, Germany) was also used.

### Generation of NP1 stable transfected NSCLC cells

NP1 plasmid DNA was inserted into the site of the mammalian vector pcDNA3.1(-) (Invitrogen Corporation, CA, USA) to generate pcDNA3.1(-)-NP1 plasmid constructs. The NP1 plasmid constructs, including a pcDNA3.1(-) empty vector control, were individually transfected into the NP1 negative cell line, H460. Stable transfections were carried out using FuGENE HD™ transfection reagent (Roche Diagnostics Ltd., UK). Cells (3 × 10^5^) were cultured in their respective supplement-free medium and transfected with either 1 μg pcDNA3.1(-)-NP1 or pcDNA-3.1(-) (control vector) in antibiotic-free media containing 3 μL/mL FuGENE HD™ according to manufacturers’ instructions. Following transfection, cells were further incubated for 24 h at 37°C. Antibiotic selection was then carried out by treating the cells with Geneticin G418 (800 μg/mL). Following several rounds of antibiotic selection, clones were selected and characterized at the mRNA and protein levels in order to examine relative NP1 expression levels.

### *In vivo* tumor growth studies

Nude mice on a BALB/c background (CBy.CG-*Foxn1*^*nu*^) were purchased from Jackson Laboratories (Bar Harbor, MD, USA). Female mice, 10 weeks of age were utilized. Animals were housed under specific pathogen-free conditions in individually ventilated and filtered cages under positive pressure. All animal experiments were performed in compliance with Irish Department of Health and Children regulations (Licence B100/3250) and approved by the Trinity College Dublin BioResource Ethical Review Board. Mice were anaesthetized with isofluorane and injected subcutaneously on the left-hand side dorsal flank with 3 × 10^6^ H460 empty vector control cells (n = 8) or 3 × 10^6^ NP1 stable transfectant cells (n = 8). Mice were monitored and weighed weekly. Final tumor volume was recorded using digital callipers and calculated based on the equation (D1)^2^ × D2 × 0.524, where D1 is the smaller of the two diameters of the tumor measured in both directions. Experiments were terminated when the tumor volume reached 2 cm^3^. Tumors were excised and retained for further analyses. H460 cells (NP1-negative) were transfected with a NP1 plasmid to over-express this receptor for the *in vivo* component of this study.

### Statistical analysis

Statistical analysis was carried out using analysis of variance (ANOVA) with post-hoc analysis using Bonferroni multiple comparisons test, unless otherwise stated. Where the means of two data sets were compared, an unpaired Students *t*-test was used. Data is graphically represented as mean ± standard error of the mean (SEM) following three independent experiments, where p < 0.05 was considered statistically significant. All data were analyzed using GraphPad InStat™ (version 3.0) statistical software.

## Additional file

Additional file 1: Figure S1. Induction of PI3K and MAPK signaling pathways by VEGF. **Figure S2.** PI3K and MAPK signaling pathways are stimulated by VEGF in SKMES1 NSCLC cells. **Figure S3.** Confocal microscopy analysis of downstream PI3K and MAPK signaling proteins. **Figure S4.** siVEGF induces significant decreases in PI3K and MAPK signaling in SKMES1 cells. **Figure S5.** The effect of siNP1, siNP2 and KDR blockade on Akt and MAPK phosphorylation in A549 and SKMES1 NSCLC cells.
