# Correction to: Vascular endothelial growth factor is an autocrine growth factor, signaling through neuropilin-1 in non-small cell lung cancer

**DOI:** 10.1186/s12943-020-1142-8

**Published:** 2020-01-27

**Authors:** Martin P. Barr, Steven G. Gray, Kathy Gately, Emily Hams, Padraic G. Fallon, Anthony Mitchell Davies, Derek J. Richard, Graham P. Pidgeon, Kenneth J. O’Byrne

**Affiliations:** 1 Thoracic Oncology Research Group, School of Clinical Medicine, Institute of Molecular Medicine, Trinity Centre for Health Sciences, St. James’s Hospital & Trinity College Dublin, Dublin, Ireland; 2 School of Medicine, Trinity Biomedical Sciences Institute, Trinity College Dublin, Dublin, Ireland; 3 Irish National Centre for High Content Screening & Analysis, School of Clinical Medicine, Trinity College Dublin, Dublin, Ireland; 4 Cancer & Ageing Research Program, Queensland University of Technology, Brisbane, Australia; 5 Department of Surgery, Institute of Molecular Medicine, St James’s Hospital & Trinity College Dublin, Dublin, Ireland

**Correction to: Mol Cancer**

**https://doi.org/10.1186/s12943-015-0310-8**

Since the publication of this work [1] and in response to a recent query that was brought to our attention in relation to the Western Blot in Figure 1(C) for NP2, protein lysates prepared around the same time as those presented in the manuscript in question, were run by SDS-PAGE under similar experimental conditions and probed using the same primary antibodies to NP1 and NP2 that were used originally.

**Figure 1c**

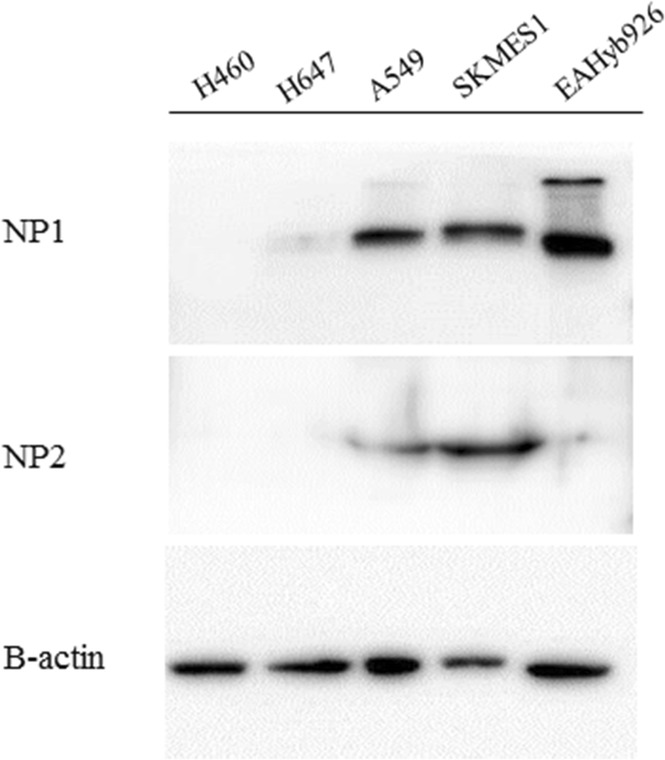



In addition, the identification of an error in the assembly of blots in Figure 4(B) for NP2 and β-actin proteins, excess protein lysates from the original experiments were run by SDS-PAGE under similar experimental conditions and probed using the same NP2 primary antibody. The results obtained were consistent with those reported in the original publication [1] and do not in any way change the data/conclusions from this particular experimental analysis.

**Figure 4b**

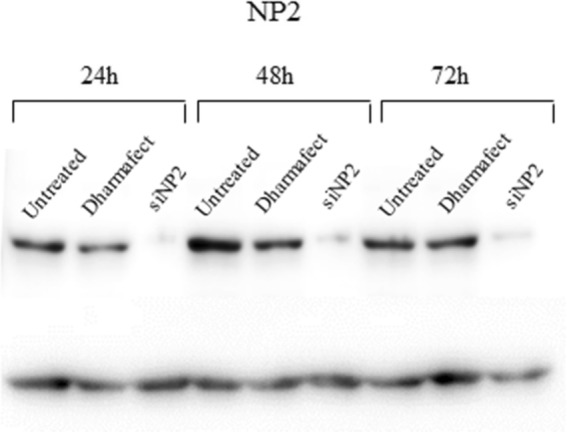



**Figure 1**

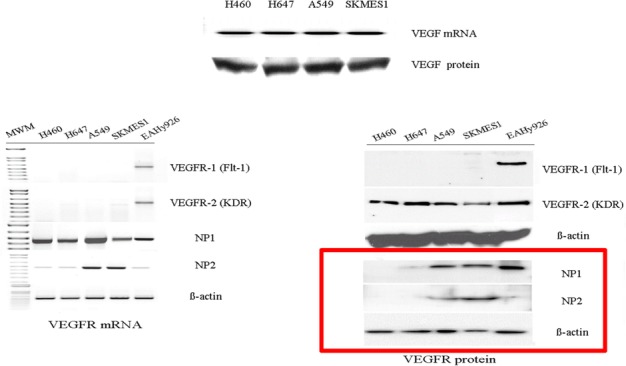



**Figure 4**


