# Supplementary material for: Vascular endothelial growth factor is an autocrine growth factor, signaling through neuropilin-1 in non-small cell lung cancer
Source: Mol Cancer. 2015 Feb 20;14:45. doi: 10.1186/s12943-015-0310-8 (PMC4392793; doi:10.1186/s12943-015-0310-8)
Supplement: Additional file 1: Figure S1. — Induction of PI3K and MAPK signaling pathways by VEGF. Figure S2. PI3K and MAPK signaling pathways are stimulated by VEGF in SKMES1 NSCLC cells. Figure S3. Confocal microscopy analysis of downstream PI3K and MAPK signaling proteins. Figure S4. siVEGF induces significant decreases in PI3K and MAPK signaling in SKMES1 cells. Figure S5. The effect of siNP1, siNP2 and KDR blockade on Akt and MAPK phosphorylation in A549 and SKMES1 NSCLC cells. [file 12943_2015_310_MOESM1_ESM.doc]

**Additional file 1**

**A. B.**

**
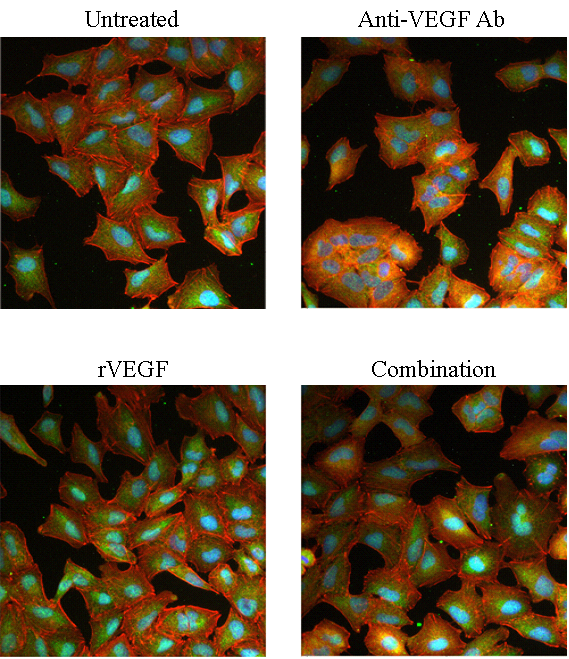

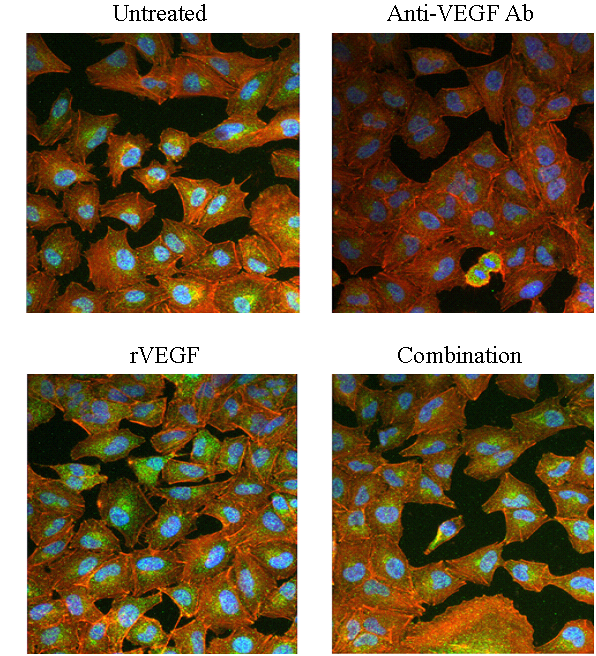
**


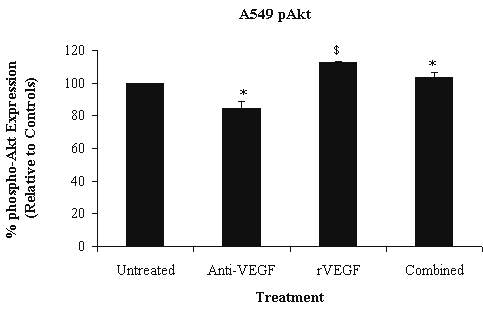

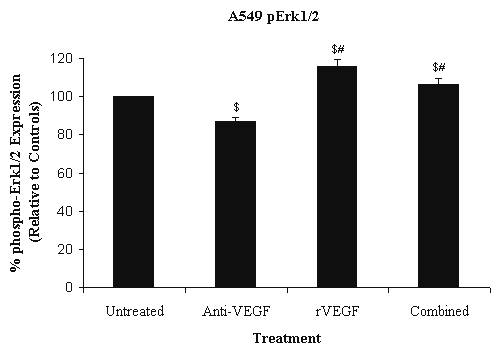


**Figure S1.** Induction of PI3K and MAPK signaling pathways by VEGF. A549 cells were treated with recombinant human VEGF (100 ng/ml), VEGF neutralizing antibodies (1 μg/ml) or both combined. Phospho-Akt (A) and phospho-Erk1/2 expression (B) expression were examined by high content analysis (HCA). Expression of the phosphorylated proteins, pAkt and pErk1/2, were quantified using IN Cell Analyzer 1000 software. Cells showing green fluorescence are representative of phosphorylated Akt and Erk1/2 proteins (white arrows) while staining of the nucleus and actin cytoskeleton are indicated as blue and red staining, respectively (Stains used: Alexa Fluor® 488, Hoechst 33342 and phalloidin, ×40 magnification). Green fluorescence intensity for pAkt and pErk1/2 proteins was quantified and expressed relative to untreated cells (^*^p<0.05, ^$^p<0.01, ^$#^p<0.001, n=3). Data are expressed as the mean ± SEM. Statistical analysis was carried out by ANOVA using the Bonferroni multiple comparison post test.


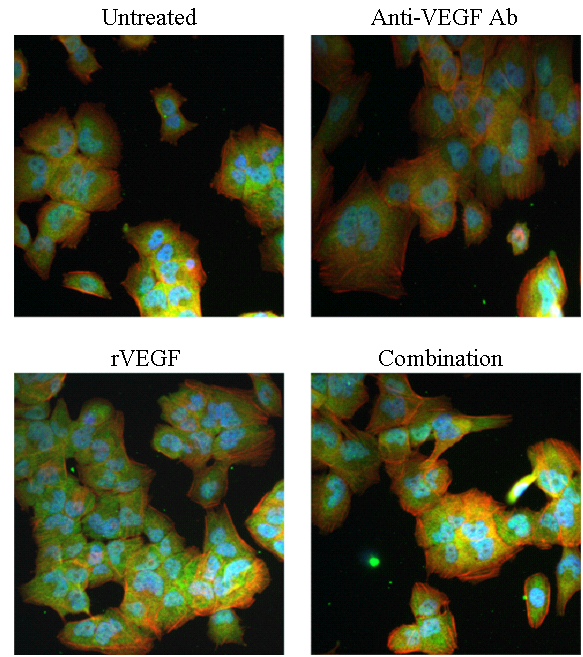

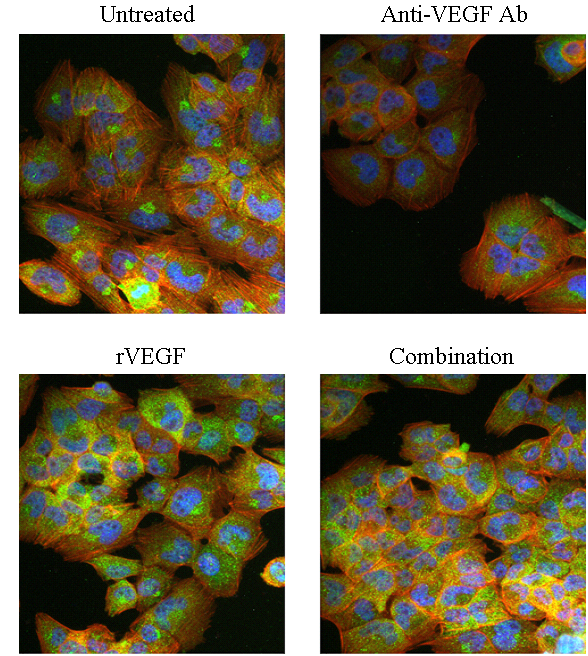


**A. B.**


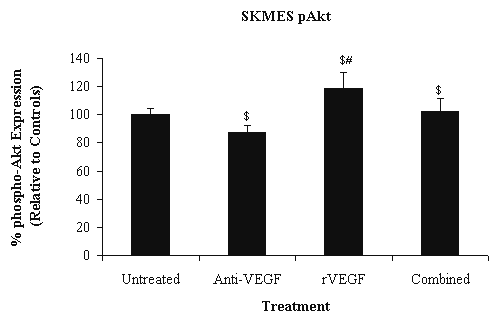

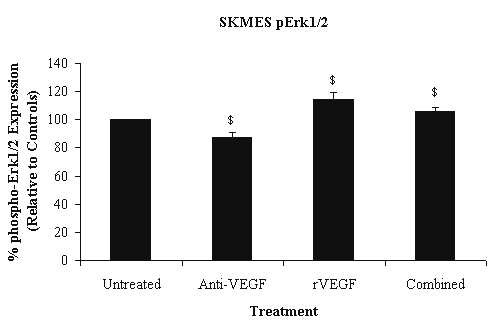


**Figure S2.** PI3K and MAPK signaling pathways are stimulated by VEGF in SKMES1 NSCLC cells. Cells were treated with recombinant human VEGF (100 ng/ml), VEGF neutralizing antibodies (1 μg/ml) or both combined. Phospho-Akt (A) and phospho-Erk1/2 (B) expression and localization were examined by high content analysis (HCA) and quantified using IN Cell Analyzer 1000 software. Representative images showing SKMES1 lung cancer cells stained for phosphorylated proteins (green), nuclear staining (blue) and actin cytoskeleton (red) are shown (Stains used: Alexa Fluor® 488, Hoechst 33342 and phalloidin, ×40 magnification). Green fluorescence intensity for pAkt and pErk1/2 proteins was quantified and expressed relative to untreated cells (^$^p<0.01, ^$#^p<0.001, n=3). Data are expressed as the mean ± SEM. Statistical analysis was carried out by ANOVA using the Bonferroni multiple comparison post test.


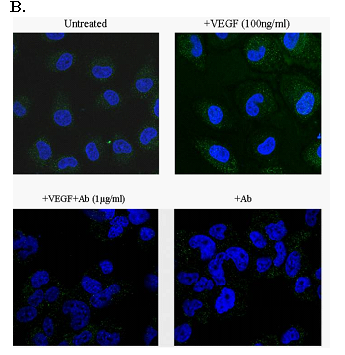


**A.**


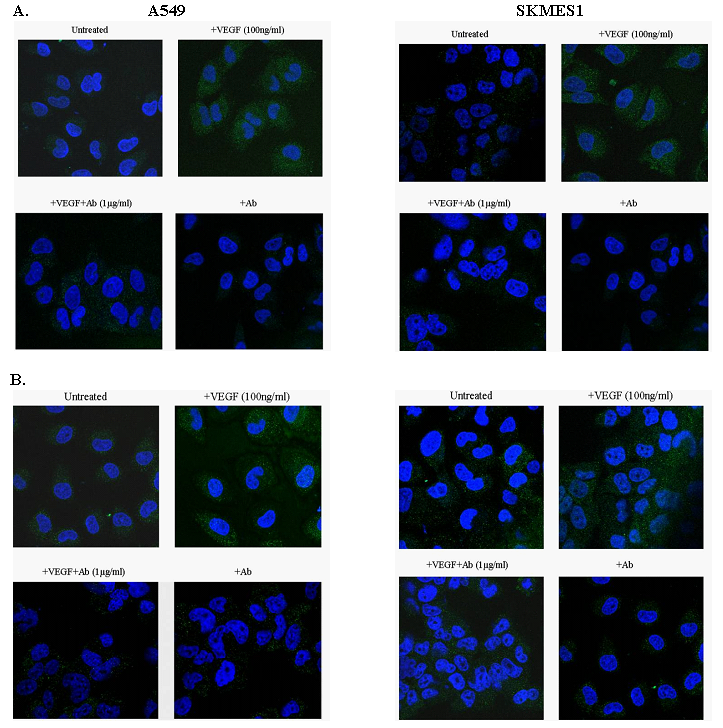

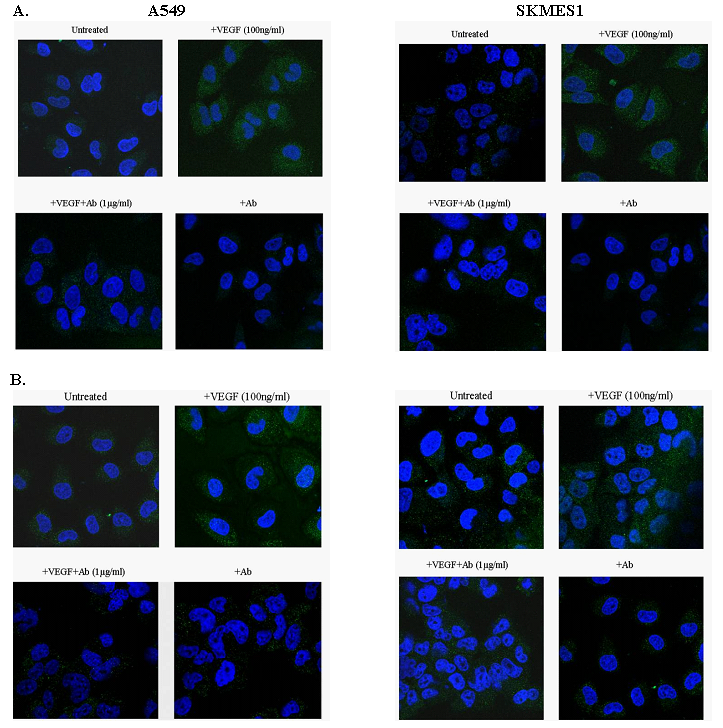


**B. C.**

**Figure S3.**


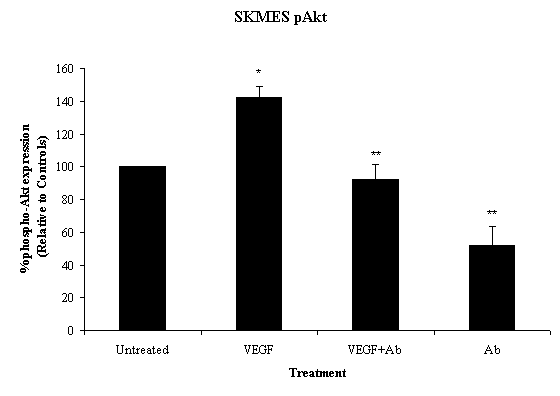

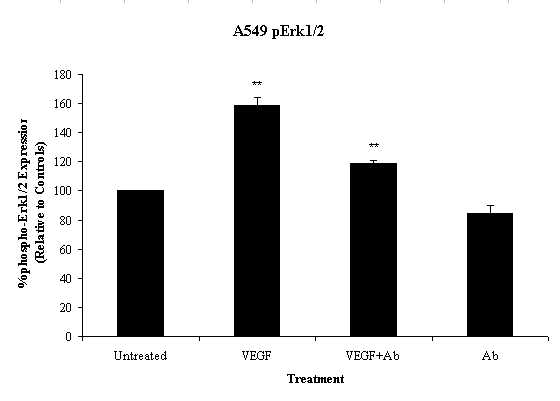

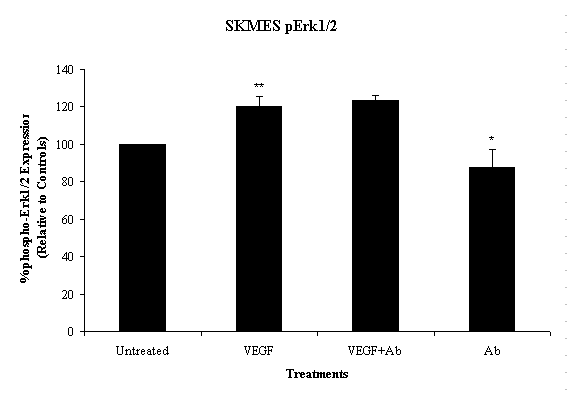


**Figure S3.** Confocal microscopy analysis of downstream PI3K and MAPK signaling proteins. Phospho-Akt and p44/p42 MAPK (Erk1/2) signaling proteins were further examined using a Zeiss LSM 510 laser scanning confocal microscope (×60 magnification). A549 and SKMES1 lung cancer cells (1×10^4^) were seeded in glass chamber slides and allowed to adhere overnight. Following serum depletion (0.5% FBS), cells were treated with recombinant human VEGF (100ng/ml) or VEGF neutralizing antibodies (1μg/ml) for 6h and fixed in 3% paraformaldehyde. Localization and expression levels of phospho-Akt were assessed in SKMES1 cells (A). p44/p42 MAPK (Erk1/2) protein expression was also examined in response to VEGF treatments in both A549 (B) and SKMES1 (C) cell lines (Stains used: Alexa Fluor® 488 and Hoechst 33342, ×40 magnification). Green fluorescence intensity for phospho-Akt and phospho-Erk1/2 proteins was quantified and expressed relative to untreated cells. Expression levels of phosphorylated proteins were also evaluated relative to treatments with VEGF vs VEGF+Ab (^*^p<0.01, ^**^p<0.001, n=3). Data are expressed as the mean ± SEM. Statistical analysis was carried out by ANOVA using the Bonferroni multiple comparison post test.


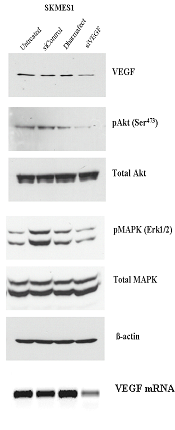


**A.**


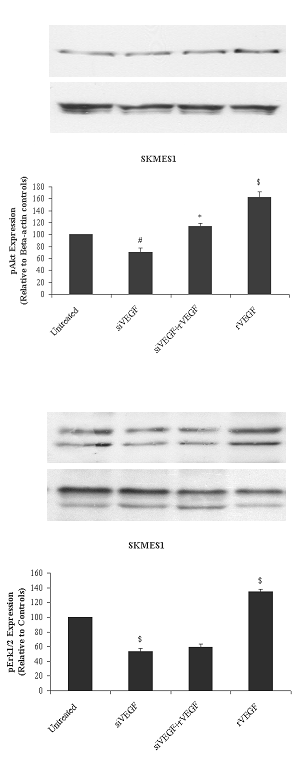


**C.**


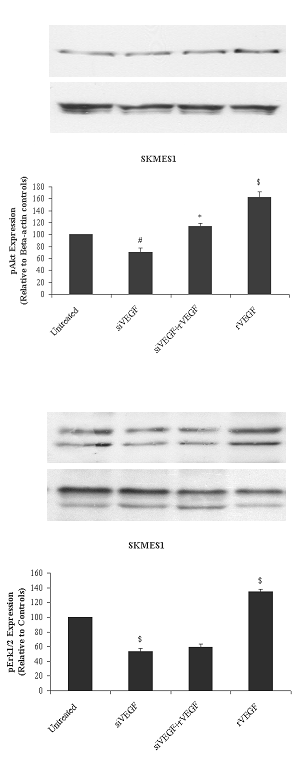


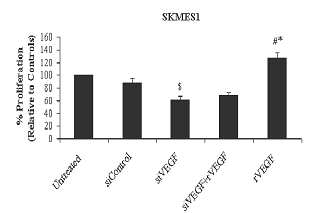

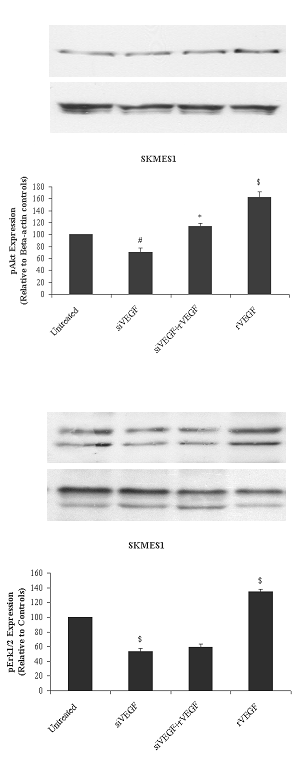


**B.**

**Figure S4.** siVEGF induces significant decreases in PI3K and MAPK signaling in SKMES1 cells. Phosphorylated Akt and Erk1/2 signaling proteins were examined by Western blot in response to siVEGF (A). VEGF mRNA expression was also assessed by RT-PCR to confirm knockdown of VEGF. Cells were treated with siRNA to VEGF (100nM) or a scrambled siRNA control for 48h, after which time, cell proliferation was measured (B) (^$^p<0.01, ^#*^p<0.001, n=3). Akt and MAPK (Erk1/2) phosphorylation was also examined in response to VEGF. Cells were treated with siVEGF either alone, or in combination with exogenous recombinant human VEGF (rVEGF) for 48h and examined by Western blot analysis (C). Densitometric analysis was used to measure phospho-Akt and phospho-Erk1/2 expression levels in SKMES1 cells. Phosphorylation levels were expressed as a percentage of untreated cells. Data are represented as the mean ± SEM. Statistical analysis was carried out by ANOVA using the Bonferroni multiple comparison post test (^#^p<0.05, ^*^p<0.01, ^$^p<0.001, n=3).


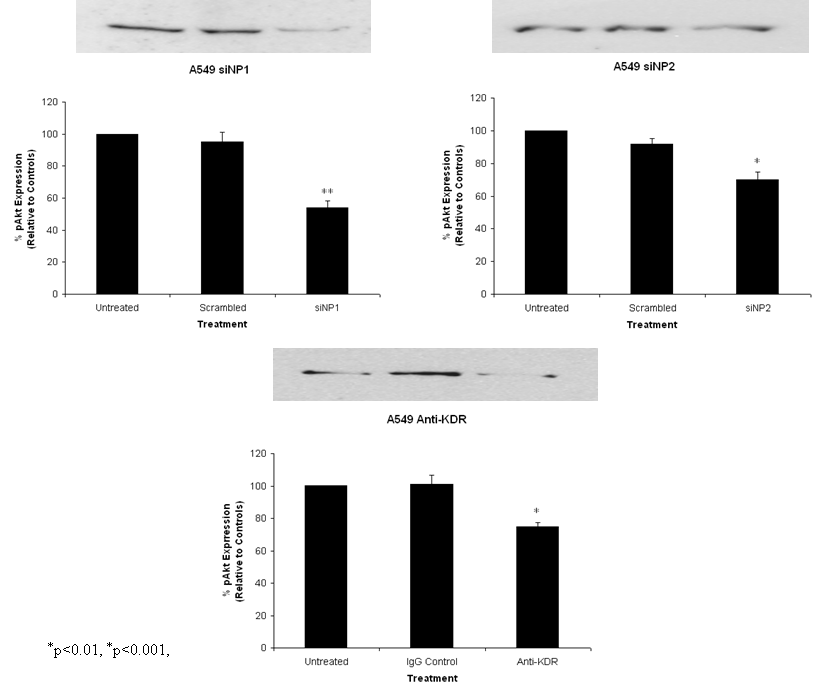


A.

**Figure S5.**


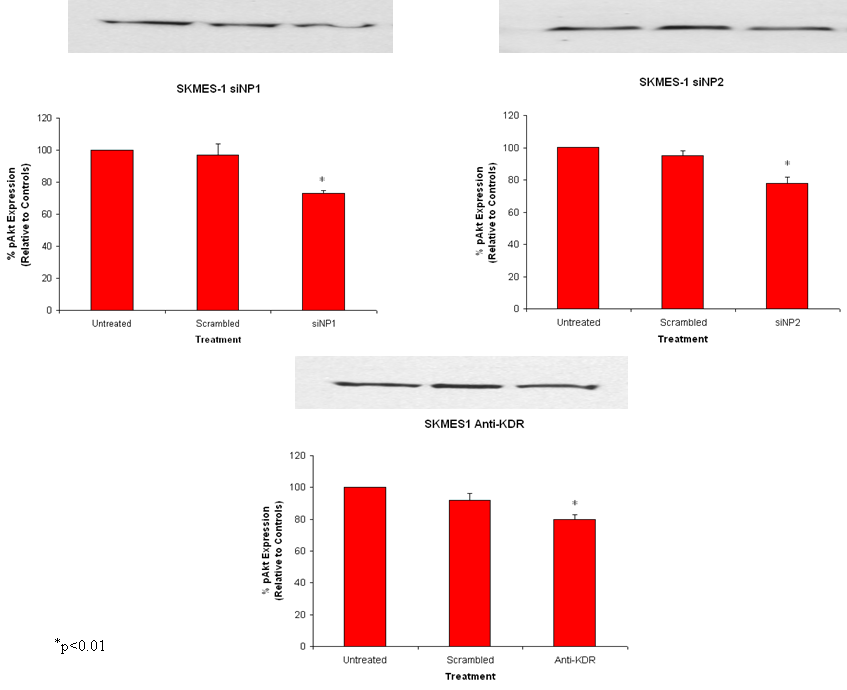


B.

**Figure S5 (continued)**

**
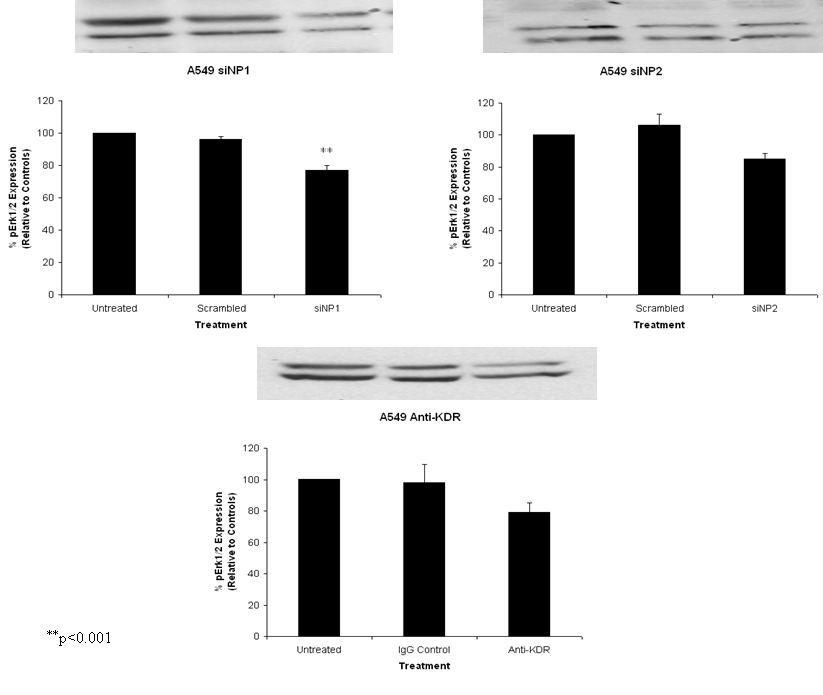
**

C.

**Figure S5 (continued)**

**
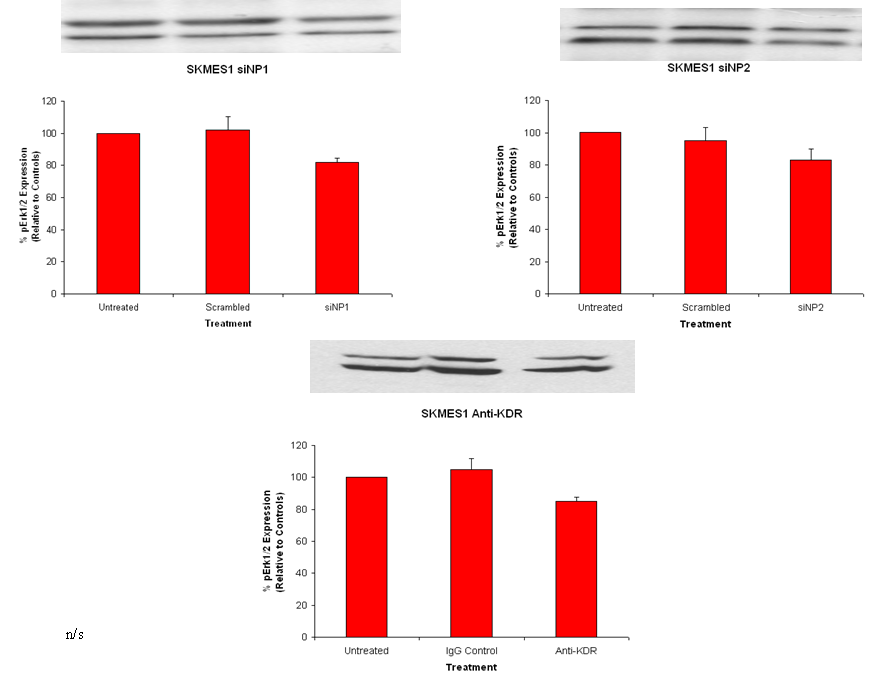
**

D.

**Figure S5.** The effect of siNP1, siNP2 and KDR blockade on Akt and MAPK phosphorylation in A549 and SKMES1 NSCLC cells. Cells were treated with siRNA or neutralizing antibody to the Neuropilin receptors NP1, NP2 and KDR, respectively. A scrambled control was included for each receptor siRNA. Expression of phosphorylated Akt (A, B) and phosphorylated Erk1/2 (C,D) proteins were measured by Western blot analysis in both cell lines. Using densitometric analysis, phospho-Akt expression levels were measured and expressed as a percentage of untreated (control) cells. Data are represented as the mean ± SEM. Statistical analysis was carried out by ANOVA using the Bonferroni multiple comparison post test (^*^p<0.01, ^**^p<0.001, n=3).
